# Excretion of Dietary Cow's Milk Derived Peptides Into Breast Milk

**DOI:** 10.3389/fnut.2019.00025

**Published:** 2019-03-12

**Authors:** Gianluca Picariello, Maristella De Cicco, Rita Nocerino, Lorella Paparo, Gianfranco Mamone, Francesco Addeo, Roberto Berni Canani

**Affiliations:** ^1^National Research Council (CNR), Institute of Food Sciences, Avellino, Italy; ^2^Department of Translational Medical Science, University of Naples “Federico II”, Naples, Italy; ^3^CEINGE Advanced Biotechnologies, University of Naples “Federico II”, Naples, Italy; ^4^Department of Agriculture, Parco Gussone, University of Naples “Federico II”, Portici, Italy; ^5^European Laboratory for the Investigation of Food-Induced Diseases, University of Naples “Federico II”, Naples, Italy; ^6^Task Force on Microbiome Studies, University “Federico II”, Naples, Italy

**Keywords:** breast milk, cow's milk proteins, food allergens, protein digestion, HPLC-MS/MS, Western blot, β-lactoglobulin, breastfeeding

## Abstract

Nanoflow-HPLC-tandem mass spectrometry (MS/MS) was used to analyze the peptide fraction of breast milk samples collected from a single non-atopic donor on different days (10 samples) after receiving an oral load of cow's milk (by drinking 200 mL of bovine milk). In addition, breast milk was sampled from the same lactating mother over a 6-h period at five time points after drinking cow's milk. We aimed to trace the intra-individual variability and to define a time profile of the excretion of dietary peptides into breast milk. Overall, 21 peptides exclusively originating from both bovine caseins and whey proteins with no match within the human milk proteome were identified in the breast milk samples. These peptides were missing in the breast milk obtained from the mother after a prolonged milk- and dairy-free diet (three samples). The time course of cow's milk-derived β-Lg f(125–135) and β-casein f(81–92) in breast milk was determined from the MS ion intensity of the peptide signals. No intact cow's milk gene products were detected by HPLC-MS/MS analysis and Western blotting with anti-β-Lg antibody, but dot-blot analysis confirmed the occurrence of β-Lg fragments in the enriched peptide fraction of breast milk. These data suggest shifting the analytical perspective for the detection of dietary food allergens in breast milk from intact proteins to digested peptide fragments. The possible sensitization and elicitation potential or the tolerogenic properties of such low amounts of dietary peptides for the breastfed newborns remain to be explored.

## Introduction

Breast milk contains antigenically active food allergens arising from the mother's diet (1). This assertion is mainly based on immunochemical data and clinical investigations carried out in the 1980s and 90s (4–6). In those years, it had been clearly established that food allergens derived from a mother's diet can elicit clinical manifestations of adverse reactions in exclusively breastfed susceptible newborns, with an incidence rate of 0.5% (7–10). Several studies have documented the presence of variable amounts of intact food allergens in breast milk, including ovalbumin (11), gliadin (12), peanut (2, 13), and cow's milk proteins (10). Food allergens in human milk have been reported to vary over a very wide range of concentrations from just above 0.1 to >1,000 ng/mL (3), but low ppb (ng/mL) levels are probably the most realistic figures (14).

The search for cow's milk allergens in breast milk has been the subject of fervent research. Human and bovine β-casein, α_s1_-casein, and α-lactalbumin share a medium to high degree of sequence homology (53, 31, and 73% homology, respectively) and, hence, a certain immune cross-reactivity. Therefore, β-lactoglobulin (β-Lg), which is not expressed by humans, has often been monitored as a cow's milk marker. According to immunochemical data, the excretion of dietary food allergens into breast milk generally appears to be affected by marked inter- and intra-individual variability (11, 14–16). With specific concern to cow's milk allergens, exogenous β-Lg has been detected in only a limited number of mothers' milk samples, and its presence is not related to atopic or non-atopic conditions (15). In a longitudinal study carried out over the entire lactation period, 93 out of 232 (i.e., 40%) breast milk samples obtained from 25 healthy donors contained a detectable amount of dietary β-Lg (16). Only two mothers had detectable β-Lg in all their milk samples, while the milk of six mothers did not contain it on any occasion. Recently, Matangkasombut et al. (17) reported that dietary β-Lg was persistent in human milk even up to 7 days after the consumption of cow's milk.

In spite of the bulk of research carried out in regard to this topic, the evidence about dietary allergens in breast milk remains controversial, primarily because the immunochemical methods could be biased from a series of pitfalls related to the antibody recognition of the protein targets and to matrix interference effects. More recently, a few studies have attempted to detect dietary proteins in breast milk with antibody-independent methods. β-Lg in human milk should definitely be considered an artifact due to non-specific antibody cross-reactivity with endogenous human milk proteins (18, 19). However, intact bovine α_s1_-casein of dietary origin has also been detected in human milk (20) and colostrum (21) with mass spectrometry (MS)-based proteomics. Nevertheless, these findings appear to conflict with the latest observations obtained *in vivo*, which suggest that β-Lg might be partially intact even at level of the mother's intestine, whereas caseins are completely degraded in the upper gastrointestinal tract (22). On the other hand, it remains to be established whether intact or partially hydrolyzed dietary antigens could be taken up and then excreted into breast milk through routes other than the intestinal one. Such a possibility is supported by the very quick appearance of peanut allergens in breast milk, even within a few minutes after ingestion (13).

Schocker et al. (23) exploited two-dimensional electrophoresis/Western blot and MS to investigate the passage of unhydrolyzed Ara h 2 and other peanut allergens into breast milk. Interestingly, they also assayed human milk samples with competitive ELISA in order to quantify possible digestion-resistant peptides derived from peanut proteins.

For the first time, we exploited MS to detect peptides arising from the digestion of bovine casein and whey proteins excreted into the breast milk of lactating donors after an oral administration of cow's milk (24). In particular, bovine β-Lg and α_s1_-casein fragments were detected in the 12% trichloroacetic (TCA)-soluble fraction of two and one out six breast milk samples, respectively. In contrast, the β-Lg peptides identified by high-resolution/sensitivity MS escaped the competitive ELISA test performed on unfractionated milk, suggesting that they might occur with very low abundance. As expected, control milk samples obtained from six lactating women complying with strict milk- and diary-free diet were lacking cow's milk-derived peptides. The absence of intact β-Lg at detectable amounts in any breast milk samples was confirmed by sodium dodecyl sulfate-polyacrylamide gel electrophoresis (SDS-PAGE) and Western blot analysis of the 12% TCA-insoluble protein pellet. Very recently, Zhu at al. (25) obtained a highly curated inventory of non-human peptides in human milk based on MS data, and cow's milk-derived peptides were the most represented species.

In this study, we aimed to monitor the possible intra-individual variability of dietary cow's milk peptides in human milk. For this purpose, we targeted bovine milk-derived peptides in breast milk sampled from a single donor before and after opportune oral cow's milk loads on different days. In addition, breast milk was sampled from the same lactating mother over a 6-h period (five time points) after drinking cow's milk. Janssen Duijghuijsen et al. (26) elegantly highlighted the possibility that food-derived peptides of varying length might be caught by endogenous antibodies in body fluids. Thus, in addition to the 12% TCA-soluble fraction, we analyzed the tryptic digests of the insoluble protein pellet using HPLC-MS/MS.

## Materials and Methods

Ditiothreitol (DTT), iodoacetamide (IAA), guanidine, trifluoroacetic acid (TFA), ammonium bicarbonate (Ambic), Tris-base, EDTA, and other chemicals were purchased from Sigma-Aldrich (St. Louis, MI, USA). HPLC-MS-grade solvents were obtained from Carlo Erba (Milan, Italy). The bovine milk whey protein isolate used for the preparation of positive controls was obtained from Fonterra Dairy Co. via the Riddett Institute, New Zealand.

### Sampling

A total of 18 breast milk samples (5 mL) were obtained through a breast pump equipped with plastic disposable pump sets. The samples were collected during the second and third month of lactation after parturition from a non-atopic healthy donor (28 years old) who delivered at term. Breast milk samples (~5 mL) were collected over different days between 2 and 3 h after a 200-mL oral load of pasteurized bovine milk. Milk was expressed into plastic sterile tubes, mixed with a serine-protease inhibitor (Pefabloc®, Sigma, St. Louis, MI, USA, 1 mM final concentration), and immediately frozen at −20°C to prevent undesired hydrolysis.

An initial screening was performed on the breast milk samples collected from the donor over 5 different days. These samples were designated as D1, D2, D3, D4, and D5. The lactating mother complied with a strict diet that was free of cow's milk and dairy for at least 1 week. Overall, 10 samples were obtained and analyzed individually by collecting breast milk before and after feeding the baby on each sampling day. Samples of the D series were run in duplicate. Three control milk samples (baselines) were collected before the mother was given cow's milk.

After assessing the presence of bovine milk-derived peptides in breast milk, additional samples were obtained from the same mother. In this case, breast milk was collected over a 6-h period at time intervals (1, 2, 3, 4, and 6 h) following an oral load of bovine milk. These samples are designed as T0 (baseline), T1 (1 h), T2 (2 h), T3 (3 h), T4 (4 h), and T5 (6 h) and were analyzed with HPLC-MS/MS in triplicate. A skilled operator at the Department of Translational Medicine of the University of Naples “Federico II” (Italy) supervised the administration of bovine milk, sample collection, and code labeling. Prior to collecting milk samples, written informed consent was obtained from the mother.

### Peptide Extraction

Aliquots of breast milk samples were skimmed by centrifugation (3,000 × g for 15 min at 4°C), and the upper fat layer was removed with a spatula (which was repeated twice). The proteins of the skimmed milk were precipitated at 4°C with a final concentration of 12% (w/v) trichloroacetic acid (TCA) and pelleted by centrifugation (4,500 × g for 30 min at 4°C). The 12% TCA soluble peptides (250 μL) were solid-phase extracted using C18 reverse-phase spin columns (Pierce Biotechnology, Rockford, IL, USA), extensively washed with 0.1% trifluoroacetic acid (TFA), and eluted with 70% acetonitrile (AcN) containing 0.1% TFA. AcN was evaporated in a SpeedVac™ Evaporator System, and peptides were finally lyophilized.

### Tryptic Hydrolysis of the Protein Pellet

In order to remove TCA from the residual pellet, the precipitate was re-suspended in cold acetone (−20°C) and centrifuged at 4,500 × g for 30 min at 4°C three times. Finally, 5 mg of the dried protein powder was dissolved in 1 mL of a denaturing/reducing buffer (6 M guanidine HCl, 0.3 M Tris, 1 mM EDTA, 10 mM DTT, pH 8.0) and incubated at 56°C for 1 h. Afterwards, cysteines were alkylated with IAA (55 mM final concentration) for 40 min at room temperature in the dark. DTT in a stoichiometric amount was used to quench the IAA excess. The Cys-alkylated proteins (50 μL aliquots) were diluted 10-fold with 25 mM ammonium bicarbonate (AMBIC) at pH 7.8 and digested overnight at 37°C with trypsin at an enzyme-to-substrate ratio of 1:50 (w:w). The resulting peptides were purified using C18 reverse-phase spin columns and lyophilized prior to HPLC-MS/MS analysis.

### Competitive ELISA for β-Lg Fragments

Possible β-Lg-derived peptides in breast milk were assayed using the RIDASCREEN® competitive ELISA kit, which was specifically validated for the detection of hydrolyzed fragments of β-Lg (R4901, R-Biopharm AG, Darmstadt, Germany). The assay was carried out according to the manufacturer's instructions using intact bovine β-Lg as a standard. Samples were assayed in duplicate. The standard points fit a cubic spline curve that was built using the software Ridasoft Win version 1.91 (R-Biopharm AG). Unfractionated skimmed breast milk was assayed at two dilutions of 1:500 (recommended by the manufacturer) and 1:200 (v:v). To exclude matrix effects, control breast milk samples spiked with variable amounts of β-Lg were used as the positive control. The limit of detection and limit of quantification declared by the manufacturer were 2.1 and 5 ppm, respectively, with <1% cross-reactivity with caseins or other whey proteins.

### Dot-Blot and Western Blotting Detection of Hydrolyzed or Intact β-Lactoglobulin

Peptides in the 12% TCA-soluble fraction of the D-series breast milk samples were enriched and purified using C18 cartridges as described above. The 70% AcN/0.1% TFA eluate was dried in a SpeedVac apparatus, re-dissolved in 2 μL of deionized water, and entirely loaded onto a 0.22-μm-pore-size nitrocellulose membrane (Bio-Rad, Milan, Italy) for dot-blot analysis. A chymotryptic digest (1 μg) of bovine milk whey proteins dissolved in water was obtained by overnight incubation in 25 mM AMBIC at pH 7.8 and 37°C with proteomic grade chymotrypsin (Sigma) at an enzyme-to-substrate ratio of 1:50 and used as the positive control.

The 12% TCA-insoluble breast milk proteins washed with cold acetone at −20°C were assayed for intact β-Lg by Western blot analysis. Proteins were dissolved in Laemmli sample buffer at 1 mg/mL, and 10-μg aliquots were separated by SDS-PAGE using 15% acrylamide hand-cast gels (Bio-Rad Laboratories, Hercules, CA, USA). Bovine whey protein isolate was used as the positive control at approximate amounts of 2 μg or 20 pg of β-Lg for the SDS-PAGE and Western blot analyses, respectively. Gels were run in duplicate; after separation, one gel was stained using G-250 Coomassie Blue Silver staining, while the other was electroblotted onto the nitrocellulose membrane using a Trans-Blot Cell (BioRad) at 400 mA and 4°C for 1 h.

For both dot-blot and Western blotting analyses, membranes were blocked for 1 h at room temperature with 5% (w/v) bovine serum albumin (Sigma) in Tris-buffered saline solution with 0.05% Tween 20 (TBS-T) and incubated overnight at 4°C with immunoaffinity purified anti-β-Lg IgG polyclonal antibody developed in rabbit (Abcam Ltd., Cambridge, UK) at 1:10000 dilution in TBS-T. Monoclonal peroxidase-conjugated mouse anti-rabbit IgG antibody (Sigma) diluted to 1:10000 in TBS-T was applied to the membrane for 1 h at room temperature. The membrane was extensively rinsed with TBS-T (3 × 10 min) and finally with TBS (1 × 10 min) before development. Chemiluminescence reagents (ECL Plus WB reagent, GE Healthcare) and X-ray film (Kodak, Chalons/Saône, France) were used to visualize the immunoreactive peptide spots or protein bands with exposure times varying in the range of 0.5–2 min.

### HPLC-Orbitrap MS/MS

HPLC-MS/MS analyses were performed using an Ultimate 3,000 nano-flow ultra-high-performance liquid chromatography (Dionex/Thermo Scientific, San Jose, CA, USA) coupled to a Q-Exactive Orbitrap mass spectrometer (Thermo Scientific). Tryptic peptides were reconstituted in 2% AcN/0.1% formic acid, and nearly 2 μg were loaded through Acclaim PepMap 100 trap columns (75-μm i.d. × 2 cm; Thermo Scientific) using a FAMOS autosampler (Thermo Scientific). Peptides were separated using an EASY-Spray™ PepMap C18 column (2 μm, 25 cm × 75 μm) with 2-μm particles and 100-Å pore size (Thermo Scientific). The separation was done with a 2–50% gradient of B over 120 min after 5 min of isocratic elution at 2% B and a constant flow rate of 300 nL/min. Eluent A was 0.1% formic acid (v/v) in LC-MS-grade water, and eluent B was 0.1% formic acid (v/v) in AcN. MS1 precursor spectra were acquired in the positive ionization mode scanning the 1,800–300 *m/z* range with resolving power of 70,000 full width at half maximum (FWHM), an automatic gain control (AGC) target of 1 × 10^6^ ions, and maximum ion injection time of 256 ms. The spectrometer operated in full scan MS1 and data-dependent acquisition mode, selecting up to the 10 most intense ions for MS/MS fragmentation and applying a 12-s dynamic exclusion. Fragmentation spectra were obtained at a resolving power of 17,500 FWHM. Ions with one charge or more than six were excluded from the MS/MS selection. Spectra were elaborated using the software Xcalibur version 3.1 (Thermo Scientific). The MS signal intensity of selected peptides was determined by integrating the area under the peaks after an extracted ion process from the total ion current (TIC) full scan chromatogram.

### Database Search and Peptide Identification

LC-MS/MS raw data were analyzed with the Andromeda tool of the MaxQuant software package (version 1.6.2.10). The searches were taxonomically restricted to *Homo sapiens* and *Bos taurus* in the Uniprot database (updated in November 2017). Subsequently, the searches were refined using a manually constructed protein database containing the 30 most abundant cow-milk gene products inferred from proteomic-based investigations (27, 28). For the analysis of 12% TCA-soluble peptides, the search conditions included unspecific cleavage, no static modification, Met oxidation, pyroglutamic acid at N-terminus Gln, and Ser/Thr phosphorylation as variable modifications.

For the analysis of the protein pellets, trypsin was selected as the proteolytic enzyme with up to two missed cleavages, and carbamidomethyl-cysteine was included as a static modification. The analysis of peptides from the pellet was also repeated under conditions of non-specific cleavage in order to identify possible bovine milk-derived peptides arising from the mother's digestion and associated with breast milk antibodies. In all cases, the mass tolerance value was 5 ppm for the precursor ion and 10 ppm for MS/MS fragments. Peptide Spectrum Matches (PSMs) were filtered using the target decoy database approach with an e value of 0.01 peptide-level false discovery rate (FDR), corresponding to a 99% confidence score. Since there is significant overlap between human and bovine milk protein sequences, it was necessary to refine the data further by identifying peptides that matched both proteomes. Peptides that were an exact match to both a bovine and a human sequence were removed from the final list of results. Only peptides originating from the relatively abundant bovine milk proteins were included in the list of identified bovine peptides.

## Results

Due to an intense activity of endogenous proteases, human milk is intrinsically rich in oligopeptides (29), which interfere with the MS-based detection of trace amounts of possible foreign peptides arising from the mother's diet. Although the search for cow's milk-derived peptides is practically restricted to relatively few abundant proteins (casein and major whey proteins), there is no option to predict which peptides might be excreted into breast milk due to the substantial lack of cleavage specificity for protein degradation during the pre- and post-absorptive phases in the mother's body. Thus, the analytical complexity of the system requires a combination of “deep” and “untargeted” peptidomics that can be addressed only using state-of-art advanced peptidomic approaches. Such a complexity would also provide a plausible reason for why little or no attempts have been made to detect food allergen-derived peptides in human milk so far.

### Dietary Cow's Milk Peptides in Breast Milk

On average, more than 1,200 peptides derived from endogenous proteins were identified by HPLC-MS/MS in the 12% TCA-soluble peptide fraction of each breast milk sample (not shown), as already reported in our previous work (24). However, the repertory of the endogenous peptides exhibited a significant intra-individual variability, as clearly observed when comparing the chromatographic profiles. As expected, most of these peptides were hydrolytic fragments of human β-casein and other caseins, while only a few of them were derived from α-lactalbumin, which is the most abundant protein of mature human milk. In fact, α-lactalbumin is relatively resistant to proteolysis under the chemical conditions of milk because of the compact folding in comparison to the looser structure of caseins. In contrast, a significantly high number of peptides arose from human lactoferrin, osteopontin, butyrophilin, lactadherin, and polymeric-Ig-receptor (24). Since we were interested in identifying peptides of dietary origin, we refined our search using a database containing the 30 most abundant cow-milk gene products. Considering the D1–D5 samples and excluding the exact sequence matches between bovine and human species, 21 peptides were exclusive products of the bovine milk proteins, while they had no counterpart within the human milk proteome (Table 1). In terms of the presence of cow's milk peptides, the pairs of samples collected before and after baby feeding were practically undistinguishable. For this reason, the pairs of D1–D5 samples were not designed with different labels. Thus, for example, in Table 1, D1 collectively designates the breast milk samples obtained on the first collection day either before or after feeding the baby. Cow's milk peptides excreted into breast milk were chiefly from β-casein and β-Lg, although traces of other caseins (α_s1_- and κ-casein) and lactoferrin were identified at a very high degree of confidence as well (FDR 1%, p < < 0.01).

**Table 1:** LC-MS/MS-based identification of cow's milk specific peptides in D samples.

**Mass ([M+H]^**+**^)**	m/z*	**Charge**	**Error (ppm)**	**sequence**	**bovine parent protein**	**Uniprot PAN**	**Position**	**Samples**
943.4886	472.2480	2	0.2	GGVSLPEWV	α-lactalbumin	P00711	19–27	D2
1117.5184	559.2626	2	−0.9	TPEVDDEALE	β-lactoglobulin	P02754	125–134	D3, D4, D5
1245.5844	623.2961	2	0	TPEVDDEALEK	β-lactoglobulin	P02754	125–135	D1, D3
1455.7210	728.3638	2	0.9	VEELKPTPEGDLE	β-lactoglobulin	P02754	43–55	D1, D3, D5
1681.8912	841.4492	2	1.0	VEELKPTPEGDLEIL	β-lactoglobulin	P02754	43–57	D3, D4, D5
1204.6100	602.8086	2	−0.1	YVEELKPTPE	β-lactoglobulin	P02754	42–51	D1, D3
978.5103	489.7588	2	−0.3	YVEELKPT	β-lactoglobulin	P02754	42–49	D3
1041.5438	521.2755	2	0.3	VEELKPTPE	β-lactoglobulin	P02754	43–51	D3
1108.6145	554.8112	2	−0.5	ENLLRFFVA	α_s1_-casein	P02662	18–26	D2, D3
964.4454	482.7266	2	−1.6	ESTVATLED	κ-casein	P02668	140–148	D3
1120.6379	560.8226	2	0.3	LPQNIPPLTQ	β-casein	P02666	70–79	D1, D3, D4, D5
1007.5517	504.2795	2	−0.3	PQNIPPLTQ	β-casein	P02666	71–79	D1, D3
882.4759	441.7418	2	0.6	QPLPPTVM	β-casein	P02666	149–156	D1, D2, D3, D4
1320.7497	660.8785	2	0.2	PVVVPPFLQPEV	β-casein	P02666	81–92	D2, D3, D4, D5
1221.6865	611.3469	2	0.1	PVVVPPFLQPE	β-casein	P02666	81–91	D1
1047.6558	524.3315	2	−0.3	VLPVPQKAVP	β-casein	P02666	170–179	D1
1024.4378	512.7228	2	0.8	ESPQTHYY	lactoferrin	P24627	86–93	D1, D2, D3
1021.4944	511.2511	2	−0.5	GSNFQLDQL	lactoferrin	P24627	101–109	D3, D4
1149.5528	575.2800	2	−0.6	GSNFQLDQLQ	lactoferrin	P24627	101–110	D3, D4
901.5460	451.2766	2	−0.5	VAVVKKGSN	lactoferrin	P24627	95–103	D4
1166.4905	583.7489	2	1.4	YEEYLGTEY	lactoferrin	P24627	657–665	D2, D4, D5

Error has been calculated after the MaxQuant re-calibration. *In the cases of peptides recurring in more than one sample, the m/z values are the closest to the theoretical ones.

Interestingly, β-casein-derived peptides are proline-rich sequences, which are hardly hydrolyzed by gastrointestinal enzymes (30). β-Lg-derived peptides belong to two protein domains, namely the 42–57 and 125–135 regions, which are known to survive the gastrointestinal digestion relatively well (31–33). β-Lg f(125–135) is also partly translocated across models of the intestinal epithelium, suggesting that it could be up-taken and reach the portal blood circulation (34). β-Lg f(42–57) and its shortened form, β-Lg f(42–54), were already identified in the breast milk of two lactating women in a previous study (24). It is worth noting that a second donor that had been initially enrolled in the study was excluded because traces of cow's milk peptides were found in her control baseline breast milk, especially peptides derived from β-casein. Upon specific inspection of the alimentary record, we realized that she had inadvertently eaten dairy products a few hours before milk collection. Importantly, the set of exogenous peptides significantly changed among the different samples at both qualitative and quantitative levels since some of them recurred in several samples at varying intensity, while others such as κ-casein f(140–148) appeared only once in sample D3. Their size varied between 6 and 17 residues, although longer fragments or whey proteins-derived S-S cross-linked heterodimeric peptides escaping the MS identification might have a chance of occurring as well.

### Variability of the Peptide Profile

We traced the time course of the presence of peptides with dietary origin in breast milk by analyzing the peptide fraction of samples collected at several time points (samples T0–T5). The peptides exclusive to cow's milk detected in these breast milk samples are listed in Table 2. Some of the peptides identified were the same or belonged to the same protein regions detected in the D samples, while others were exclusive of the two set of samples, thus confirming the variability of exogenous species excreted into breast milk.

**Table 2:** Identification of cow's milk specific peptides in T samples.

**Mass ([M+H]^**+**^)**	***m/z***	**Charge**	**Error (ppm)**	**Sequence**	**Bovine parent protein**	**Uniprot PAN**	**Position**	**Samples**
943.4874	472.2476	2	−1.1	GGVSLPEWV	α-lactalbumin	P00711	19–27	T3, T4
1204.6097	602.8084	2	−0.1	YVEELKPTPE	β-lactoglobulin	P02754	42–51	T1, T2
1117.5184	559.2626	2	−0.9	TPEVDDEALE	β-lactoglobulin	P02754	125–134	T1, T2, T3
1245.5843	623.2957	2	−0.1	TPEVDDEALEK	β-lactoglobulin	P02754	125–135	T1, T2, T3, T4
1108.6148	554.8113	2	−0.5	ENLLRFFVA	α_s1_-casein	P02662	18–26	T1, T2
784.4086	392.7082	2	−0.1	IESPPEI	κ-casein	P02668	153–159	T2
1120.6374	560.8223	2	1.1	LPQNIPPLTQ	β-casein	P02666	70–79	T1, T2
882.4680	441.7376	2	0.4	QPLPPTVM	β-casein	P02666	149–156	T2, T3, T4
1320.7497	660.8786	2	0.2	PVVVPPFLQPEV	β-casein	P02666	81–92	T1, T2, T3, T4
1840.9879	614.3341	3	0.1	LPPTVMFPPQSVLSLSQ	β-casein	P02666	151–167	T2, T3

**Figure 1:**
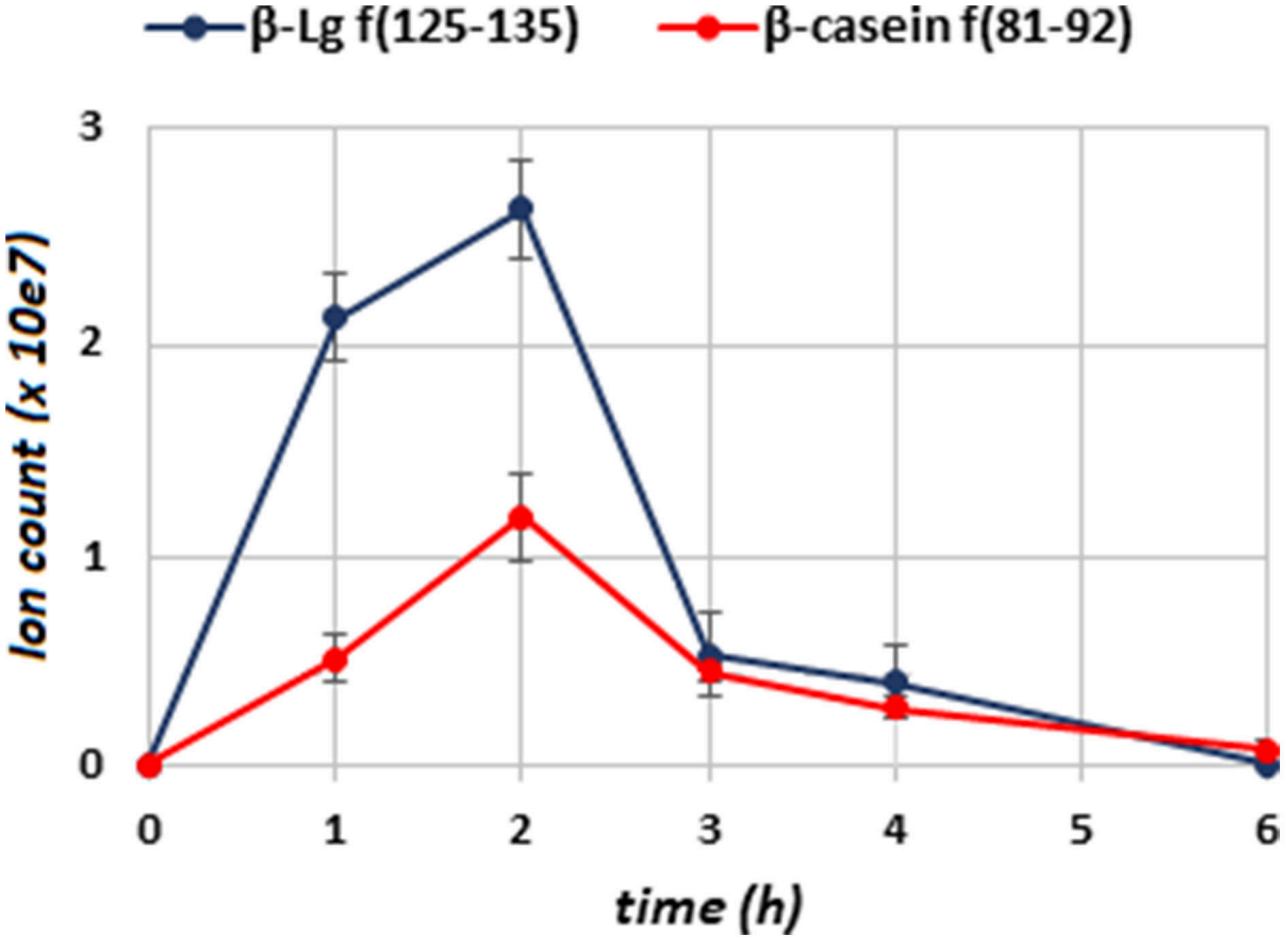
Time course of the β-Lg f(125–135) (blue line) and β-casein f(81–92) (red line) in breast milk, determined by the MS ion intensity of the peptide signals in samples of the T series. Error bars are the standard deviation of triplicate determinations.

**Figure 2:**
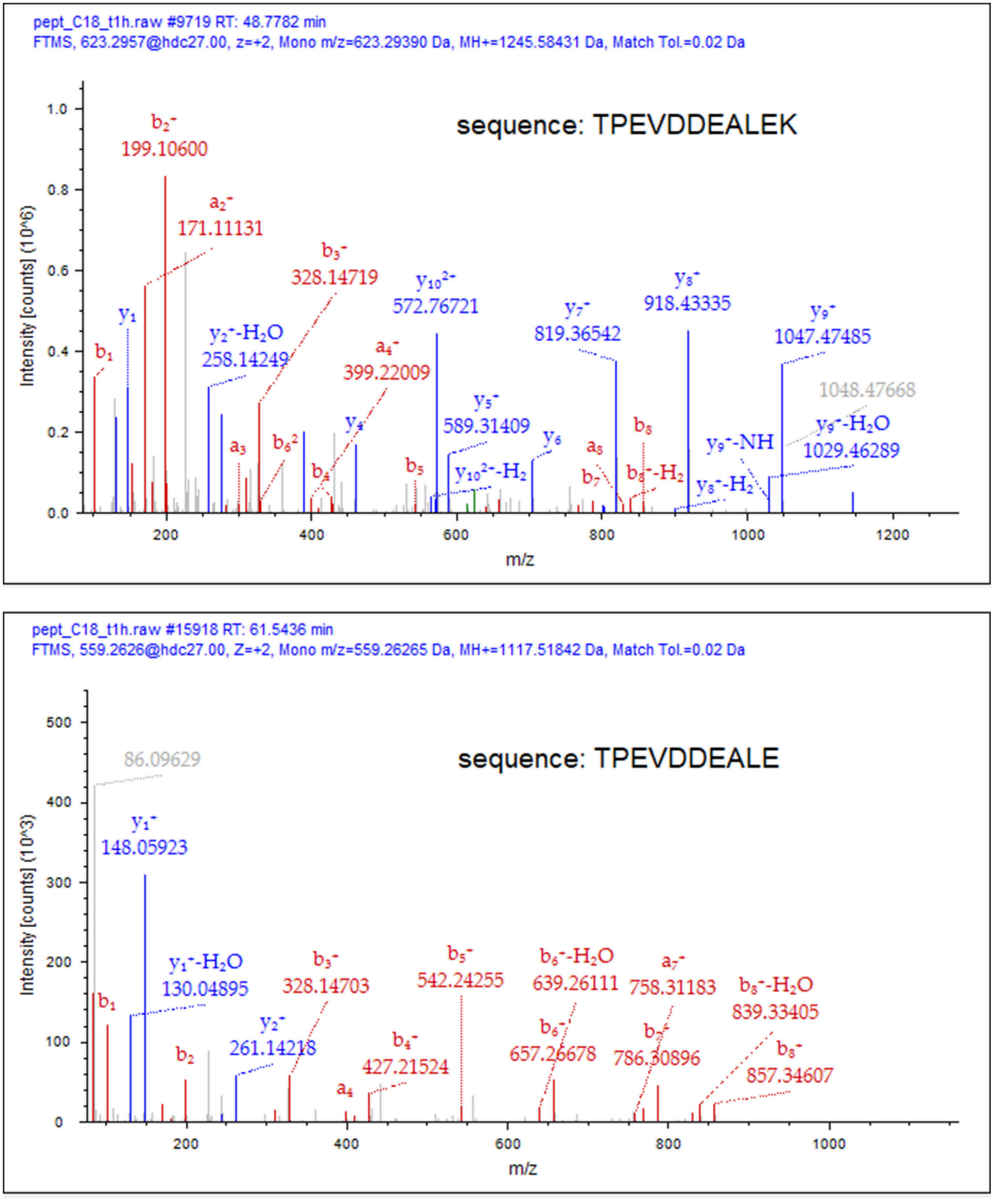
Comparative MS/MS spectra of β-Lg f(125–135) and β-Lg f(125–134) in T2 sample. Matching fragments of the a-, b- and y-series have been assigned using the Proteome Discoverer 2.1 software (Thermo Scientifics). The removal of the C-terminal Lys residue drastically alters the ionization efficiency and the fragmentation scheme of the peptide.

To deduce the kinetics of the appearance and disappearance of the dietary peptides in breast milk, we selected two sequences, namely β-Lg f(125–135) and β-casein f(81–92), which were detected over the longest time interval (T1–T4) and gave the most abundant signals within the cow's milk derived components. After individuating the position of the peptides along the chromatograms through the MS/MS fragmentation, the proximate abundance of these peptides was estimated by extracting the ions from the TIC chromatogram and integrating the area of the MS signal from the full scan run, at their specific retention time. These values were plotted vs. time since the administration of bovine milk (Figure 1). Dietary peptides were already detected at 1 h (T1) after the consumption of cow's milk and peaked after 2 h, whereas none of them was detectable 6 h (T5) after the oral load. Perhaps, carboxypeptidases mediated the time course of the conversion of the peptide β-Lg (f125–135) into β-Lg (f125–134) upon the removal of the C-terminal lysine. It was not possible to monitor this conversion from a quantitative perspective because the removal of the lysine residue significantly alters the ionization efficiency of the peptides and also drastically affects their fragmentation scheme. The MS/MS spectra of the two peptides are compared in Figure 2, which evidences that the y-series was prevalent in the fragmentation of β-Lg f(125–135) (amino acid sequence: TPEVDDEALEK), whereas the b-series dominated the fragmentation spectrum of β-Lg f(125–134) (sequence: TPEVDDEALE).

**Figure 3:**
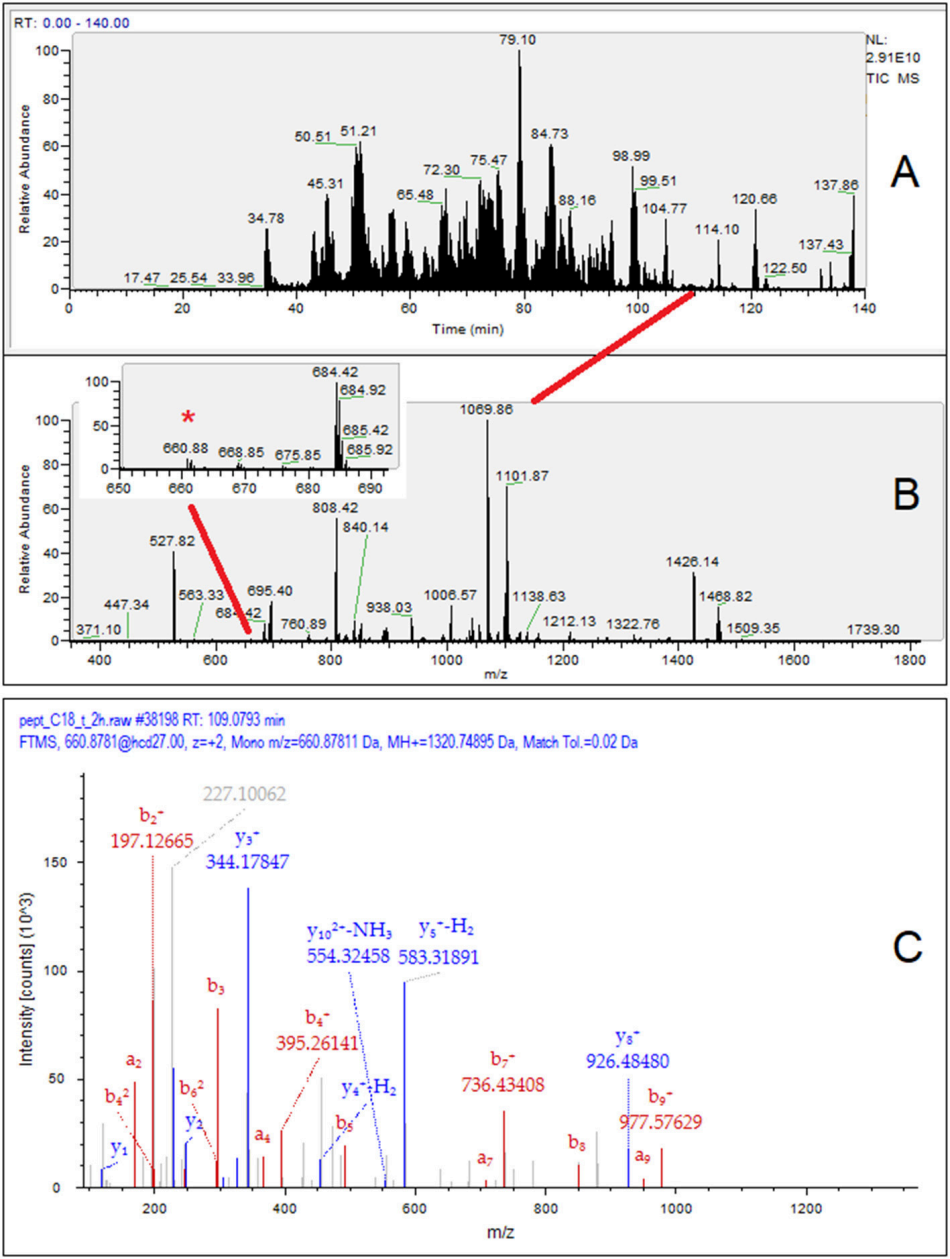
Exemplificative workflow for the identification of the peptide β-casein f(81–92) in the T2 breast milk sample. **(A)** HPLC MS/MS TIC chromatogram of the 12%TCA soluble peptides; **(B)** MS1 spectrum at retention time (RT) 109.0–109.2 min. The inset in panel B shows a magnified view containing the m/z 660.88 signal (*); **(C)** MS/MS spectrum of the m/z signal 660.88 with assignment of the β-casein f(81–92) signal fragments. The peptide of dietary origin is a minor MS signal contained in a very low-abundance chromatographic peaks, so indicating its scant relative amount in breast milk.

For illustration, the identification workflow of the β-casein f(81–92) is presented in Figure 3, which includes the TIC chromatogram of the HPLC-MS/MS analysis relevant to sample T2 (Figure 3A), the MS spectrum at 109.0–109.2 min (Figure 3B), and the MS/MS fragmentation spectrum of the ion m/z 660.8781 (Figure 3C). The inset of Figure 3B highlights the very-low-intensity MS signal (ion intensity 2.71 × 10^6^) of β-casein f(81–92). In terms of the ion count, the dietary peptide with the highest intensity, β-Lg f(125–135), did not exceed 6.1 × 10^6^, while average intensity of noise approached 4.0 × 10^5^.

Therefore, the signals of milk-derived peptides was at least 3.5–4 orders of magnitude (5 × 10^3^-10^4^-fold) less intense than that of the most abundant endogenous peptides of breast milk (intensity in the 1.7–2.9 × 10^10^ range), which seriously challenges the dynamic range of the MS method.

These findings demonstrated that peptides derived from the maternal diet occurred at a very low abundance in comparison to the endogenous peptides of breast milk, which in turn represent only a fraction of the protein/polypeptide content of human milk.

The quantitative figures of this study represent a gross estimation of the exogenous peptide abundances. An accurate relative quantification among the T-series samples could be achieved by labeling the peptides with stable-isotope tags generating low-mass reporter fragments (e.g., iTRAQ method). The absolute quantification of the dietary peptides would require a dedicated study with appropriate standards and should be performed sample-by-sample, due to the composition variability of the polypeptide fraction of human milk (25). In spite of the traditional classification of dietary proteins as “fast” (whey proteins) and “slow” (caseins) based on their relative digestion and absorption rates (34), the early-appearing peptides arose from both caseins and whey proteins. This suggests that hydrolytic fragments from the two protein families could be rather quickly digested, distributed, and excreted into breast milk.

### Dot-Blot Analysis

**Figure 4:**
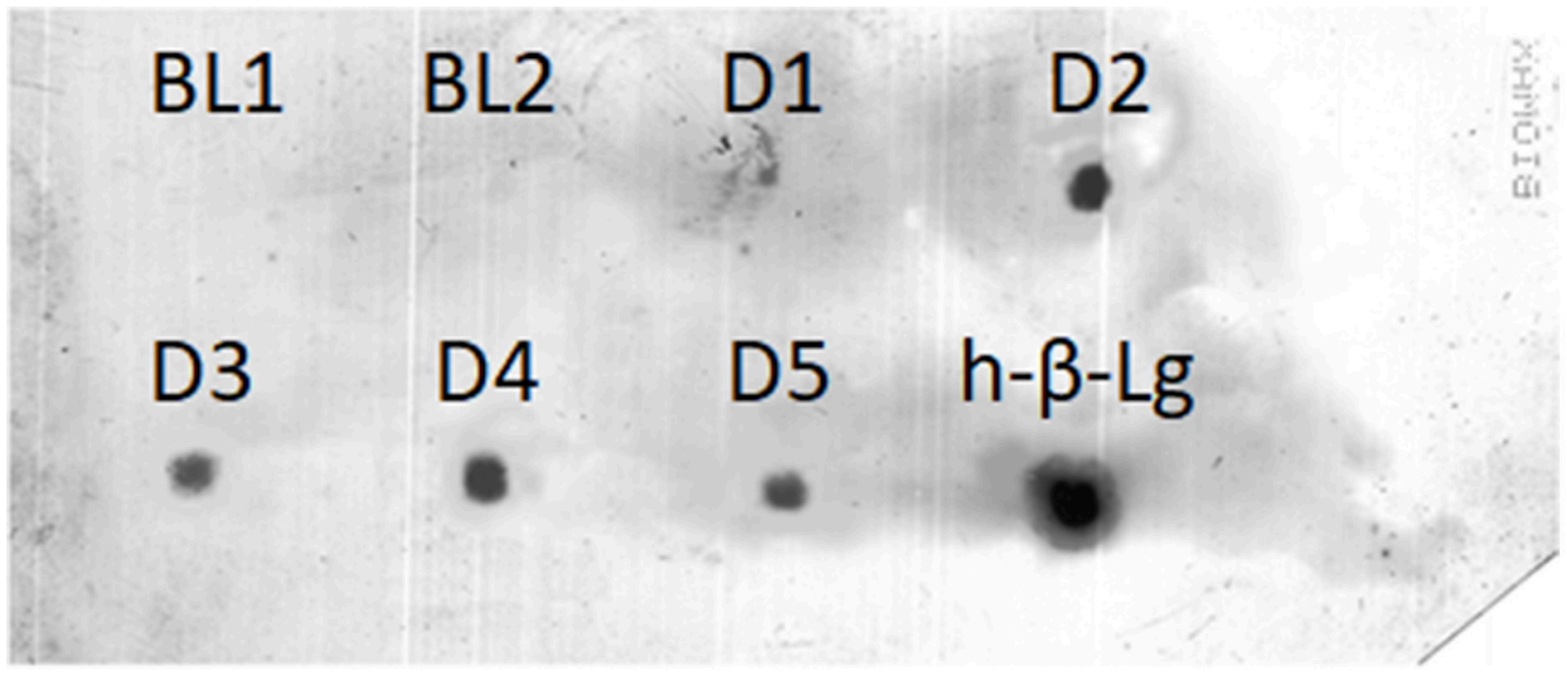
Dot-blot detection of immunoreactive β-Lg peptides in the 12% TCA soluble fraction of D-series breast milk samples. Baseline (BL) samples were peptide extracts from breast milk obtained before the cow's milk oral load. H-β-Lg was a chymotryptic digest of β-Lg used as the positive control.

β-Lg-derived peptides in the enriched and purified 12% TCA-soluble peptide fraction of the D-series breast milk samples were targeted by dot-blot analysis. Peptide extracts of all the samples were clearly recognized by the anti-β-Lg antibody, similarly to the chymotryptic digest of bovine whey proteins used as the positive control (Figure 4). In contrast, the baseline breast milk samples collected before the oral load of cow's milk were not immunoreactive, thereby excluding possible cross-reactivity with peptides originating from endogenous milk proteins. The intensity of the immunoreactive spots could not be correlated to the number and ion intensity of the β-Lg-derived peptides detected by HPLC-MS/MS because possible immunoreactive β-Lg polypeptides might escape the MS analysis.

### Competitive ELISA

In the attempt to quantify β-Lg peptides, we assayed all breast milk samples with a competitive ELISA test that was specifically developed to determine both native β-Lg and derived peptides in processed food matrices. The standard β-Lg solutions fit a cubic spline curve, in line with the manufacturer's guidelines. None of the breast milk samples contained detectable β-Lg peptides at the declared limit of detection of 2.1 ppm, confirming our previous results (24). The sensitivity of the competitive ELISA was most likely inadequate to detect the β-Lg peptides, which plausibly occurs at low ppb amounts according to a gross estimation based on the relative MS signal intensities. Less diluted breast milk samples (i.e., 1/50 and 1/100) assayed by ELISA gave inconsistent results due to matrix interference. In general, ELISA-based methods can lead to false-positive and false-negative results when applied to the determination of trace allergens in complex food matrices, which emphasizes the need for analytical methods that do not rely on antigen-antibody recognition (35).

### Searching for Cow's Milk Peptides in the Protein Pellet

Dietary polypeptides in body fluids, especially blood or breast milk, might be taken up by antibodies or associated with proteins through non-specific interactions. Such an outcome might also explain why the ELISA methods failed to detect those protein fragments revealed by MS (26). In order to target possible intact cow's milk proteins or dietary peptides associated with carrier antibodies or proteins in breast milk, the 12% TCA-insoluble protein pellet was Cys-reduced/alkylated in denaturing buffer (6 M guanidine), and the resulting tryptic digests were analyzed by HPLC-MS/MS. The database containing cow's milk protein sequences was searched using both trypsin cleavage specificity or non-specific cleavage. On average, almost 400 endogenous proteins per sample were identified with high confidence (1% FDR, e < 0.01), which have already been described in human milk (36, 37). However, no peptides ascribable to cow's milk proteins occurred in breast milk samples except for the β-Lg f(125–135) peptide, albeit at trace amounts. This peptide was detected by MS at very low intensity in only sample D4. Most likely, the presence of this peptide fragment was the result of an occasional non-specific association with breast milk proteins.

Very recently, Zhu et al. (25) identified a set of cow's milk-derived peptides—especially from bovine α_s1_-casein—in all the analyzed human milk samples. Milk was sampled from mothers who were not exposed to any dietary intervention. Based on the identified peptides, the authors hypothesized the occurrence of intact bovine α_s1_-casein in human milk, which is in line with previous findings (21). However, the estimated level of intact bovine α_s1_-casein (0.006 μg/μL) appears very high, even much more than the value indicated as the upper amount reported so far for dietary proteins in breast milk (3). In contrast, we were not able to detect any of the bovine α_s1_-casein peptides generated by tryptic digestion of the protein pellet, despite a careful visual inspection of the LC-MS/MS runs (targeted ion extraction) specifically aimed at detecting the sequences identified by Zhu et al. (25) in all human milk samples analyzed. This finding further supports the inter-individual variability of the excretion of dietary peptides into breast milk.

### Western Blotting for the Detection of Intact β-Lg

**Figure 5:**
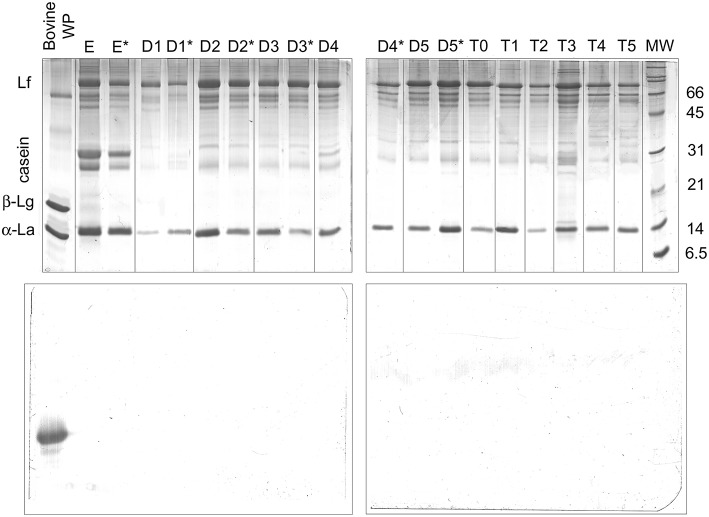
SDS-PAGE electrophoresis (upper panels) and anti-β-Lg Western blotting (lower panels) of protein pellets from D1–D5 and T0–T5 breast milk samples. D samples were collected before (w/out*) and after (*) a baby's suck. E samples are from the donor excluded from the study. Bovine whey proteins were the β-Lg positive control. For the Western blotting analysis, the estimated amount of β-Lg in the control was 20 pg. Lf, lactoferrin; β-Lg, β-lactoglobulin; α-La, α-lactalbumin.

The possible occurrence of dietary intact β-Lg was evaluated by SDS-PAGE and parallel Western blot analysis (Figure 5). Interestingly, α-lactalbumin (14 kDa) and lactoferrin (76 kDa excluding glycosylation) dominated the protein fraction of breast milk samples. Intact caseins migrating at the apparent molecular weight of 31 kDa appeared as minor bands in the SDS-PAGE pattern, most likely due to extensive proteolysis. However, the breast milk samples obtained from the different donor who was excluded from the study (lanes E and E^*^) exhibited more intense bands of intact caseins, which confirms the great inter-individual variability of the human milk protein fraction. A bovine whey protein extract was used as the positive control, with estimated amounts of β-Lg of 2 μg for the SDS-PAGE analysis and ~20 pg for the ECL reagent-based detection.

The estimated limit of detection of β-Lg by the Western blot was abundantly below 1 pg (24). As expected, β-Lg was clearly detected in the whey protein extract with both of the analyses. In contrast, no band ascribable to intact β-Lg or derived immunoreactive polypeptides was detected in any of the breast milk samples. These findings confirmed the substantial absence of intact β-Lg in the analyzed samples.

## Discussion

The findings of this study confirmed our previous outcomes suggesting that dietary peptides rather than intact allergens could be excreted into breast milk (24). This work has enlarged our previous repertoire of cow's milk-derived peptides in breast milk, which included the analysis of human milk samples obtained from 12 donors. The peptide sequences identified were relatively short and, therefore, their effective involvement in the immune response is questionable. On the other hand, there is a possible occurrence of additional large-sized protein fragments or even whey protein-derived disulfide cross-linked peptides, which are hardly evidenced by MS-based methods. To this purpose, T2 probably contained a 30-residue-long bovine κ-casein peptide, namely κ-casein f(135–165), which was identified by the search engine with a relatively low identification score. This peptide was not included in Table 2, because the MS/MS did not contain a pattern of fragment ions sufficient for a definitive attribution, though the measured molecular weight matched the theoretical one (Δmass < 1 ppm).

The panel of cow's milk peptides appeared strongly variable in the milk of a single donor, which was collected on several days as well as over a 6-h time course. These observations should be confirmed by extending the study to a higher number of samples. Unfortunately, only a handful of lactating mothers agreed to enroll in such a study, and the milk of only a few of them contained detectable cow's milk-derived peptides. Consistently, β-Lg has been reported to occur in 62.5 (38) or 75% (39) of milk samples from a small cohort of lactating mothers, which showed the appearance and disappearance as well as the variability of abundance over the 15-h sampling.

The presence of dietary peptides in breast milk provides indirect evidence that fragments of food proteins can survive the gastrointestinal digestion, pass into the bloodstream, and distribute to peripheral organs. The inter- and intra-individual variability has already been reported by several studies, although they were carried out by immunochemical methods in terms of the excretion of intact allergens or derived large-sized polypeptides into breast milk (2, 14, 39, 40). The contributions of various factors to the fluctuation and to kinetics appearance of dietary peptides in breast milk remain to be established, such as inherent variable digestion capacity, the co-consumption of other foods and their structure, altered intestinal permeability, and transitory leakage at the level of the mammary epithelium.

The clinically evident adverse reactions of predisposed newborns to food ingested by the mother represents proof that dietary peptides in breast milk are immunologically active. It has been amply demonstrated that upon the mother's abstinence from milk and dairy products, atopic manifestations against cow's milk proteins in previously sensitized breastfed infants disappear (9). On the other hand, the onset of cow's milk allergy in infants is not correlated with the presence of bovine proteins in breast milk (9, 38, 41). Although capable of eliciting allergic reactions, it is not known whether the small amounts of food antigens present in maternal milk contribute to prime sensitization or whether sensitization occurs upon exposure through different routes (chiefly the skin). In fact, many children with food allergies experience their first adverse reaction upon the first known ingestion of the offending food, indicating that a previous occult sensitization had occurred. In this work, we did not detect large cow's milk-derived polypeptides or intact proteins in breast milk. This finding contrasts with part of the previous literature (20, 21). However, on the basis of our results, we can exclude that very tiny amounts of undigested allergens might be excreted into breast milk. The very rapid appearance of food allergens in the mother's biological fluids, including blood and breast milk has been explained with an uptake through the skin or oral mucosa, which also would justify the very rapid onset of allergy symptoms in some breastfed newborns (13). Nevertheless, the evidences that dietary proteins could reach undigested the biological fluids are still weak. It has been suggested that food allergens might be processed and degraded at the lysosomal level by mucosal cells before being adsorbed and distributed (42). Supposedly, the predominance of the skin or gastrointestinal penetration routes of dietary antigen might also depend on nature of a meal or structure of the food. For example, peanuts must be chewed, while milk is quickly swallowed and has a very short time of persistent contact with the oral mucosa. Regardless, the effect of breastfeeding on the development of adverse reactions to foods or allergic diseases in newborns is still controversial, substantially because the mechanisms regulating the thin boundaries between the acquisition of tolerance and sensitization to food allergens in early infanthood are still poorly defined. Breastfeeding has commonly recognized protective effects against allergenic manifestations in children, especially those with a familial atopy (43, 44). However, these effects are still debated in the context of food allergies. Recent evidence indicates that the route of exposure to food allergens in early life has a key role in determining sensitization or oral tolerance (45). In particular, the low amount of foreign antigenic proteins in breast milk seems to be tolerogenic rather than causing early sensitization to food allergens. This suggests that lactating mothers should not observe an elimination diet during pregnancy and lactation as a strategy to prevent childhood allergies in newborns who are at risk of atopy.

## Conclusions

Breast milk could contain trace amounts of peptides resulting from the digestion of dietary proteins eaten by the lactating mother. The profile of cow's milk-derived peptides in breast milk after the mother consumes cow's milk appears affected by inter- and intra-individual variability. The plausible content of the food-derived peptides varies within the ppb range or less, and their identification was possible only upon enrichment of the peptide fraction of breast milk and high-resolution/sensitivity MS-based analysis. Dot-blot using anti-bovine β-Lg antibody also revealed traces of β-Lg in the TCA-soluble fraction after peptide enrichment. In contrast, no intact cow-milk protein allergens were detected in the breast milk. The presence of dietary peptides suggests that the analytical perspective should be shifted with respect to the large number of studies reporting on the immunochemical detection of intact dietary food allergens in breast milk. However, the possibility that large-sized food-derived polypeptides occur in breast milk cannot be ruled out. The eventual amount of these foreign polypeptides might be out of reach for common immunoassays (such as ELISA) when applied to unfractionated breast milk. The possible sensitization/elicitation potential or the tolerogenic properties of such low amounts of dietary peptides remain to be explored. Similarly, the accurate quantification of exogenous peptides in breast milk should be performed with properly designed quantitative proteomic techniques, over a large number of lactating mothers.

## Author Contributions

RB and GP conceived the study. RN and LP sampled human breast milk. MDC, RN, and LP carried out the laboratory experiments. GP, GM, and MDC performed the mass spectrometry experiments. GP and FA wrote the paper. RB revised the text. All the authors have read and approved the final version of the manuscript.

### Conflict of Interest Statement

The authors declare that the research was conducted in the absence of any commercial or financial relationships that could be construed as a potential conflict of interest.
